# A reagentless electrochemical immunosensor for sensitive detection of carcinoembryonic antigen based on the interface with redox probe-modified electron transfer wires and effectively immobilized antibody

**DOI:** 10.3389/fchem.2022.939736

**Published:** 2022-08-08

**Authors:** Jing Zhang, Luoxing Yang, Jie Pei, Yanzhang Tian, Jiyang Liu

**Affiliations:** ^1^ Shanxi Bethune Hospital, Shanxi Academy of Medical Sciences, Tongji Shanxi Hospital, Third Hospital of Shanxi Medical University, Taiyuan, China; ^2^ Tongji Hospital, Tongji Medical College, Huazhong University of Science and Technology, Wuhan, China; ^3^ Key Laboratory of Surface & Interface Science of Polymer Materials of Zhejiang Province, Department of Chemistry, Zhejiang Sci-Tech University, Hangzhou, China

**Keywords:** electrochemical determination, immunosensor, label free, sensitive detection, carcinoembryonic antigen

## Abstract

Convenient and sensitive detection of tumors marked in serum samples is of great significance for the early diagnosis of cancers. Facile fabrication of reagentless electrochemical immunosensor with efficient sensing interface and high sensitivity is still a challenge. Herein, an electrochemical immunosensor was easily fabricated based on the easy fabrication of immunoassay interface with electron transfer wires, confined redox probes, and conveniently immobilized antibodies, which can achieve sensitive and reagentless determination of the tumor marker, carcinoembryonic antigen (CEA). Carboxyl multi-walled carbon nanotubes (MWCNTs) were firstly modified with an electrochemical redox probe, methylene blue (MB), which has redox potentials distinguished from those of redox molecules commonly existing in biological samples (for example, ascorbic acid and uric acid). After the as-prepared MB-modified MWCNT (MWCNT-MB) was coated on the supporting glassy carbon electrode (GCE), the MWCNT-MB/GCE exhibited improved active area and electron transfer property. Polydopamine (PDA) was then *in situ* synthesized through simple self-polymerization of dopamine, which acts as the bio-linker to covalently immobilize the anti-CEA antibody (Ab). The developed immunosensor could be applied for electrochemical detection of CEA based on the decrease in the redox signal of MB after specific binding of CEA and immobilized Ab. The fabricated immunosensor can achieve sensitive determination of CEA ranging from 10 pg/ml to 100 ng/ml with a limit of detection (LOD) of 0.6 pg/ml. Determination of CEA in human serum samples was also realized with high accuracy.

## Introduction

Cancer nowadays seriously threatens human health owing to high morbidity and mortality. Early diagnosis and treatment of cancer is the most effective way to reduce mortality (Ashraf et al., 2022a; Asif et al., 2022). Tumor markers (TM) are effective indicators for early diagnosis of cancer and monitoring of therapeutic effects (Sadighbayan et al., 2019; Farzin et al., 2020; Zheng et al., 2021; Laraib et al., 2022). It is well known that carcinoembryonic antigen (CEA) is the most sensitive tumor marker (Liu et al., 2015; Pirsaheb et al., 2019). Thus, convenient and highly sensitive detection of CEA in serum is crucial for the early diagnosis of cancers (Jozghorbani et al., 2021). Until now, the detection of CEA commonly uses immunoassay methods based on signals of radiation, time-resolved fluorescence, chemiluminescence, or electrochemiluminescence (Liu et al., 2015; Pirsaheb et al., 2019; Jozghorbani et al., 2021). These detection strategies usually suffer from expensive instruments, special reagents, and skilled operations. In comparison with other detection strategies (for example, optical technology) (Zhang et al., 2020; Zhao et al., 2020; Deng et al., 2021; Wan et al., 2021; Zhu et al., 2022a), electrochemical sensing has attracted extensive attention because of its fast detection speed, a simple instrument, easy integration, and miniaturization (Asif et al., 2018; Ashraf et al., 2019; Aziz et al., 2019; Liu et al., 2020; Ashraf et al., 2021a; Ashraf et al., 2021b; Deng et al., 2021; Zhang et al., 2022a; Zhu et al., 2022b; Wei et al., 2022; Zhong et al., 2022; Zhou et al., 2022). The development of a novel electrochemical immunosensor for simple and highly sensitive detection of CEA is of great significance.

The detection modes in electrochemical immunoassay are usually divided into two categories (Farzin et al., 2020; Zheng et al., 2021; Laraib et al., 2022). One is based on the free electrochemical probes in solution. Usually, the specific recognition between antigen and antibody will affect the diffusion of the electrochemical probe to the electrode surface or reduce its electron transfer on the electrode interface. However, the use of a solution-phase probe might compromise the detection efficiency due to the increased complexity of the operation and possible contamination of the target system. The other detection mode is based on the immobilized electrochemical probes, which can realize reagentless detection with convenient operation. Fabrication of reagentless electrochemical immunoassay with convenient antibody immobilization and effective signal amplification is highly desirable.

The immobilization of antibody (Ab) is crucial for the construction of an efficient immuno-recognitive interface and the subsequent detection performance. Biomimetic modification of the micro/nano interface is commonly effective to immobilize antibodies. Inspired by the structure of marine mussels, polydopamine (PDA) chemistry has been used as a simple and versatile approach for the (bio) functionalization of materials (Alfieri et al., 2022). On the one hand, PDA coating can improve the hydrophilicity and biocompatibility of materials. Polydopamine is the product of the self-polymerization of dopamine (DA) under alkaline conditions with air or oxygen as the oxidant. Usually, PDA can form and stably combine on the surface of almost all materials (for example, ceramics, semiconductors, metals, and even synthetic polymers) (Samyn, 2021; Alfieri et al., 2022; Prabhu et al., 2022). On the other hand, polydopamine has secondary reactivity and can be directly used as the bio-linker for covalent immobilization of recognitive antibodies (Ding et al., 2016; Huang et al., 2020; Li et al., 2021). It has been proven that under alkaline conditions, the catechol groups in the polydopamine matrix can be oxidized to dopaquinone, which can undergo Schiff base reaction or Michael addition reaction with nucleophilic groups (for example, amine or thiol groups). Thus, PDA can be applied to directly immobilize antibodies since proteins contain −NH_2_ or −SH groups. This antibody immobilization process based on PDA is convenient with no need for complex reaction conditions or equipment.

**FIGURE 1:**
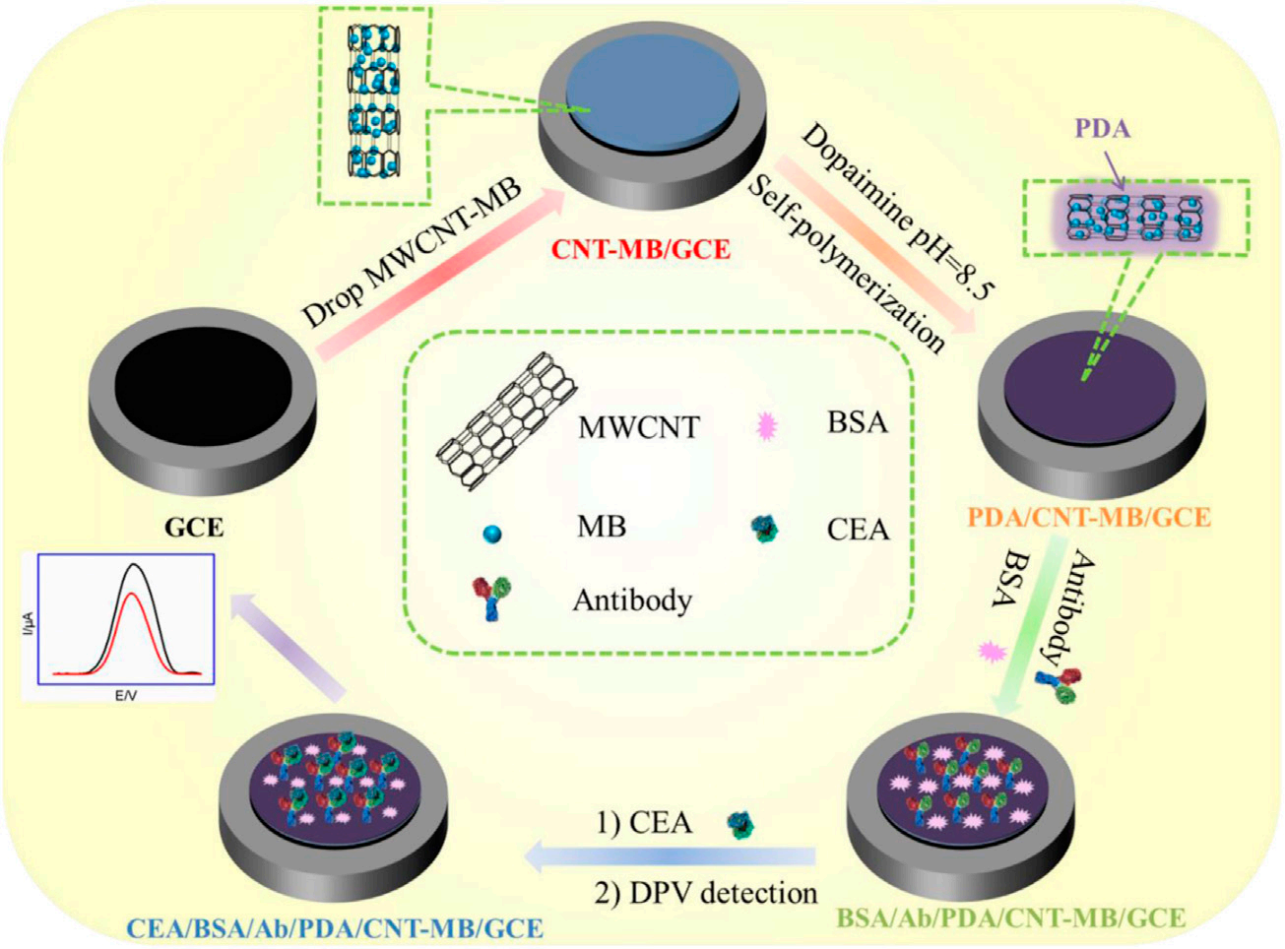
Schematic illustration for the fabrication of immunoanalysis interface and the following label-free electrochemical determination of CEA.

In this work, a simple strategy for the convenient construction of electrochemical immunosensor and its application for reagentless and sensitive determination of CEA are demonstrated. As shown in Figure 1, methylene blue (MB) was selected as the electrochemical redox probe because its redox potential is distinguishable from that of common redox molecules (for example, ascorbic acid and uric acid) in biological samples. Owing to good electrical conductivity, carboxyl multi-walled carbon nanotube (MWCNT) was employed as one-dimensional quantum wires to improve the performance of the fabricated electrochemical sensor (Mazloum-Ardakani et al., 2013; Benvidi et al., 2015; Benvidi et al., 2016; Asif et al., 2018; Yazdanparast et al., 2019; Ashraf et al., 2020; Ashraf et al., 2021b; Iftikhar et al., 2021). After MWCNT was modified by MB, the obtained composite (MB-MWCNT) was applied to modify the glassy carbon electrode (GCE), so that electron transfer wires and immobilized probes were integrated on the surface of GCE. Under alkaline conditions, polydopamine (PDA) was synthesized *in situ* through simple self-polymerization of dopamine in alkaline conditions. Subsequently, the covalent immobilization of the CEA antibody (Ab) was easily achieved by using PDA as the biological linker. When CEA specifically recognized Ab, the redox signal of MB was weakened because the antigen-antibody complex hindered the electron transfer on the electrode surface. Based on this principle, sensitive, and reagentless, electrochemical detection of CEA was achieved. The advantages of the fabrication of the electrochemical method lie in the easy fabrication of an immunoassay interface with electron transfer wires, confined redox probes, and conveniently immobilized antibodies, which can be used as a universal platform to construct an electrochemical sensor for reagentless determination of tumor marker.

## Materials and methods

### Chemicals and materials

Carcinoembryonic antigen (CEA), anti-CEA (Ab), prostate specific antigen (PSA), and carcinoma antigen 125 (CA125) were purchased from Beijing KEY-BIO Biotech Co., Ltd. (China). S100 calcium-binding protein β was purchased from Proteintech (China). Bovine serum albumin (BSA), 3-hydroxytyramine hydrochloride (DA), and sodium phosphate dibasic dodecahydrate (Na_2_HPO_4_·12H_2_O) were obtained from Aladdin (China). Sodium dihydrogen phosphate dehydrates (NaH_2_PO_4_·2H_2_O) were obtained from Macklin (China). Phosphate buffer solution (PBS) was prepared using Na_2_HPO_4_ and NaH_2_PO_4_. Methylene Blue trihydrate (MB) was obtained from Tianjin Yongda Chemical Reagent Co. Ltd. (China). Carboxyl multiwall carbon nanotubes (MWCNTs) were purchased from Chengdu organic chemicals Co. Ltd. (China). Human blood serum (healthy man) was provided by Shanxi Bethune Hospital (Taiyuan, China). All other chemicals were of analytical grade and used without further treatment. Ultrapure water (18.2 MΩ cm) was prepared by the Mill-Q system and used throughout the work.

### Measurements and instrumentations

Scanning electron microscopy (SEM) images were obtained at an acceleration voltage of 2 kV on a SU8010 microscope (Hitachi, Japan). All electrochemical investigations including cyclic voltammetry (CV), electrochemical impedance spectroscopy (EIS), and differential pulse voltammetry (DPV) were performed on the electrochemical workstation (Autolab PGSTAT302N, Metrohm, Switzerland) in the laboratory with constant temperature (25°C) and humidity (75%). The traditional three electrode system was applied in all electrochemical experiments. Briefly, bare or modified GCE was used as the working electrode. The silver/silver chloride electrode (Ag/AgCl, saturated with sodium chloride) was adopted as the reference electrode and the platinum wire electrode was the counter electrode. Before use, GCE was sequentially polished with 0.3 μM, 0.3 μM, and 0.05 μM Al_2_O_3_ slurry, respectively. Afterward, the electrode was sonicated in ethanol and then in ultrapure water to obtain a clean mirror surface. EIS investigation was performed with the frequencies ranging from 10^4^ to 10^−1^ Hz in Fe(CN)_6_^3/4−^ (2.5 mM) containing KCl (0.1 M). The Brunauer-Emmette-Teller (BET) surface areas were measured on an ASAP 2020 analyzer (Belsorp-max, United States) by using N_2_ as the adsorption gas. X-ray photoelectron spectroscopy (XPS) measurements were carried out using Mg Ká as the excitation source (Thermo Fisher, United States). The X-ray powder diffraction (XRD) patterns were obtained on a Bruker D8 A8 Advance diffractometer with Cu K radiation (40 kV, 20 mA, λ= 1.54056 Å) (Bruker AXS, United States). The zeta potential was analyzed using a Nano Particle Analyzer (SZ-100V2, HORIBA Jobin Yvon, France).

### Preparation of methylene blue-modified carboxyl multi-walled carbon nanotubes

To synthesize MB modified carbon nanotube (MWCNT-MB), carboxyl multiwall carbon nanotubes (0.5 mg/ml) were added to MB solution (2 mg/ml). A dispersion was obtained by sonication for 30 min. Then, the dispersion was centrifuged at 14,000 rpm for 10 min to remove the supernatant. The obtained precipitate was the MWCNT-MB, which can be further re-dispersed in ultrapure water.

### Fabrication of immunosensor

The immuno-recognitive interface was a fabrication on GCE through three steps. First, 10 μL of MWCNT-MB dispersion was dropped to the surface of GCE and dried at 60°C (Yan et al., 2020). The obtained electrode was denoted as MWCNT-MB/GCE. The second step was the *in-situ* coating of PDA through self-polymerization of DA. Briefly, the MWCNT-MB/GCE electrode was immersed in DA solution (0.38 mg/ml in 0.1 M PBS, pH = 8.5). After self-polymerization of DA for 1 h, the PDA-coated electrode (PDA/MWCNT-MB/GCE) was thoroughly rinsed with ultrapure water to remove the residual DA on the electrode surface. The third step was the covalent immobilization of Ab using PDA as the linker. Commonly, the CEA antibody (30 μl, 100 μg/ml in 0.1 M PBS, pH = 7.4) was dropped on the surface of PDA/MWCNT-MB/GCE and incubated at 37°C for 1 h. After the residual antibody on the obtained electrode (Ab/PDA/MWCNT-MB/GCE) was washed with PBS (0.1 M, pH = 7.4), non-specific sites were blocked by incubation in BSA (1%, in 0.1 M PBS, pH = 7.4) for 1 h at room temperature to produce a CEA immunosensor, which was denoted as BSA/Ab/PDA/MWCNT-MB/GCE.

### Electrochemical determination of carcinoembryonic antigen

PBS (0.1 M, pH 7.4) was used as the electrolyte for the electrochemical determination of CEA. Briefly, the immunosensor, BSA/Ab/PDA/MWCNT-MB/GCE, was incubated with different concentrations of CEA (antigen) at 37°C for 60 min. The electrochemical signals of MB in the immunosensor before and after CEA binding was measured using differential pulse voltammetry. The determination of CEA in human serum (healthy women) was investigated to evaluate the application of developed immunosensor for real sample analysis. Before determination, the serum was diluted by a factor of 50.

## Results and discussion

### Strategy for facile fabrication of the immunoassay interface with electron transfer wires, confined redox probes, and conveniently immobilized antibodies

Figure 1 demonstrates the facile fabrication of the immunoassay interface and the subsequent reagentless determination of CEA based on the electrochemical signals of an immobilized mediator. As shown, electron transfer wires, immobilized redox probes, and antibodies were integrated into the immunosensor interface. Amongst, multi-walled carbon nanotubes (MWCNTs) were chosen as electron transfer wires due to their excellent electron transfer, high specific surface area, and low price. (Rivas et al., 2017; Eguílaz et al., 2019; Xie et al., 2019). Carboxylated carbon nanotubes were directly applied because of the good hydrophilicity. At the same time, methylene blue (MB), a commonly used cationic redox probe (p*K*_1_ = 11.2), was used as the electrochemical probe. (Zhang et al., 2022b; Liu et al., 2022; Prado et al., 2022; Suo et al., 2022). Since the redox peaks of MB are at a negative potential, the electrochemical interference of redox small molecules (for example, ascorbic acid-AA, uric acid-UA, dopamine-DA, tryptophan, etc) in biological samples (for example, serum) can be effectively avoided because of their positive redox potentials. MB modified MWCNT (MWCNT-MB) can be easily synthesized through their electrostatic interaction due to the negative charge of carboxylated MWCNT and the positive charge of MB.

Three steps were involved in the construction of the immunosensor. First, the MWCNT-MB composite was drop-coated on the surface of GCE to improve the active electrode area, facilitate electron transfer and introduce a redox probe. The π-π interaction or hydrophobic interactions between sp (Asif et al., 2022) carbon atoms from both GCE and MWCNTs was beneficial to the stable binding of MWCNT-MB composites. Second, a layer of PDA was *in-situ* covered on an MWCNT-MB modified electrode (MWCNT-MB/GCE) through the self-polymerization of dopamine (DA) under alkaline conditions with dissolved oxygen as the oxidant. Third, the anti-CEA antibody (Ab) was covalently linked to the PDA layer followed by the blocking of non-specific sites using bovine serum albumin (BSA). It has been shown that PDA has a structure similar to the byssin protein in marine mussels and thus has secondary reactivity. Due to the −NH_2_ or −SH groups in amino acids of proteins, PDA can be used as a linking layer to achieve covalent attachment of Ab through Michael addition or Schiff-based reactions under mild conditions.

### Characterization of carboxyl multi-walled carbon nanotubes-methylene blue nanocomposite

XRD was employed to investigate the change in the crystalline structure of MWCNT. As shown in Supplementary Figure S1 (in Supporting information, SI), two peaks located at 26.0° (d value of 3.4 Å) and 43.6^o^ (d value of 3.25 Å) were observed as (002) and (100) reflections of graphite, respectively, which are attributed to the distance between walls and interwall spacing of MWCNT (Ömür and Alanyalıoğlu, 2017; Alimohammady et al., 2019). In addition, the formation of nanocomposite with MB did not change the crystal structure of MWCNT. The changes in chemical composition between MWCNT and MWCNT-MB were further confirmed by X-ray photoelectron spectroscopy (XPS, Supplementary Figure S2 in SI). As shown, MWCNT displayed C_1s_ and O_1s_ peaks resulting from its sp (Asif et al., 2022) carbon structure and carboxylated groups. In addition to the C_1s_ and O_1s_ peaks, N_1s_ and S_2p_ peaks appeared in MWCNT-MB, proving the successful composite of MB on MWCNT. The surface potentials of MWCNT and MWCNT-MB were determined using zeta potential measurements. The zeta potential of MWCNT was −27.2 mV, which was attributed to the ionization of carboxyl groups on its surface. For MWCNT-MB, the zeta potential was −14.9 mV. The decrease in zeta potential originated from the binding of positively charged MB on the surface of MWCNT through electrostatic interaction. Brunauer-Emmett-Teller (BET) analysis revealed a specific surface area of 405.3 m^2^/g for MWCNT, which was higher than that of MWCNT-MB (337.3 m^2^/g). The slight decrease in the specific surface area might result from the increase in mass after MB binding and the possible entanglement of MWCNTs due to the electrostatic interaction with MB.

### Electrochemical characterization of the immobilized redox probes and morphology of the electrode

**FIGURE 2:**
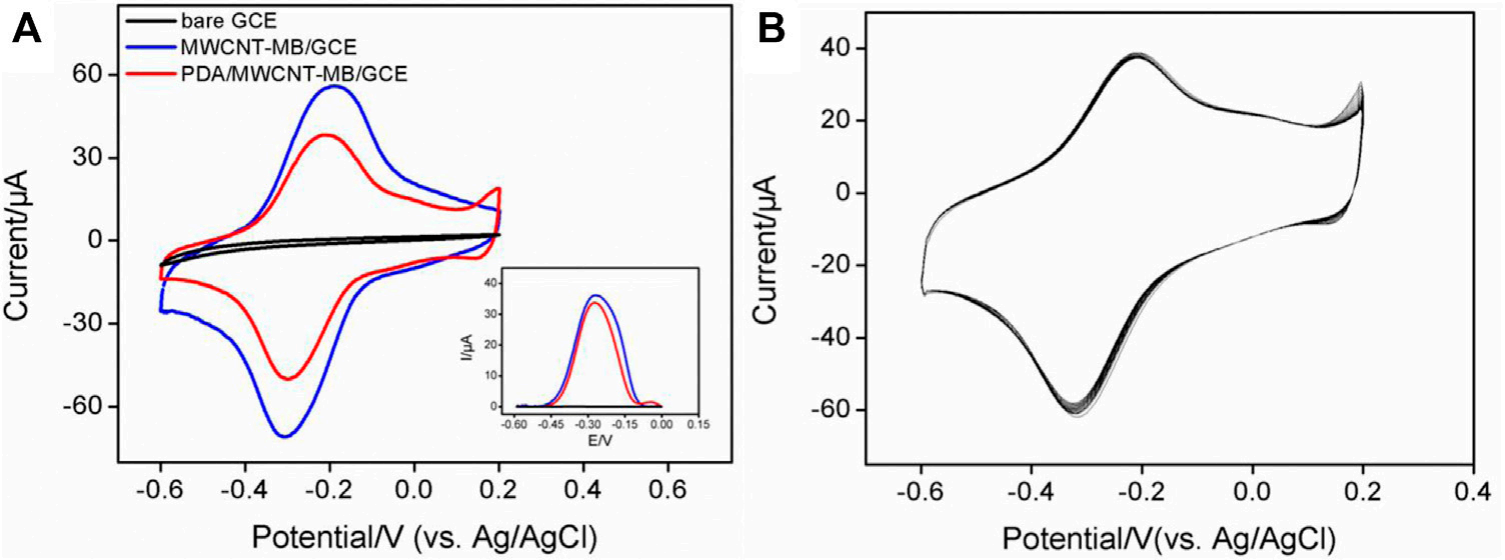
**(A)** CV and DPV (inset) curves obtained on different electrodes in PBS electrolyte (0.1M, pH = 7.4). **(B)** CV curves obtained on PDA/MWCNT-MB/GCE during continuous cyclic voltammetry scanning.

In comparison with other detection strategies (for example, optical Analysis), electrochemical detection has advantages including fast detection speed, a simple instrument, easy integration, and miniaturization. (Cui et al., 2020; Cui et al., 2021; Duan et al., 2021; Liu et al., 2022). The immobilization of redox probes on the electrode surface was investigated by detecting the electrochemical signals of MB. Figure 2A is a cyclic voltammogram of different electrodes in PBS electrolyte. As shown, no redox peak was observed on bare GCE. When GCE was modified by the MWCNT-MB composite, a pair of redox peaks was revealed on MWCNT-MB/GCE with the anodic potential (*E*_pa_) and cathodic potential (*E*_pc_) of −0.2 V and −0.32 V, respectively. These peaks were consistent with the redox potential of MB, indicating the successful immobilization of MB on the surface of the electrode. In addition, the charging current of MWCNT-MB/GCE significantly increased, proving that the introduction of carbon nanotubes effectively increased the active area of the electrode. When the PDA layer was further modified on MWCNT-MB/GCE, the redox peak current of MB slightly decreased owing to the poor conductivity of the PDA layer. These results were also proven by DPV curves in the inset of Figure 2A.

The stability of redox probes is crucial for the performance of reagentless detection. In order to investigate the stability of the immobilized probe, the prepared PDA/MWCNT-MB/GCE was continuously scanned for 20 cycles. As shown in Figure 2B, the peak current of MB hardly changed with the increase of the scanning cycles. This phenomenon indicated that MB on MWCNT could be stably immobilized on the surface of the electrode. This was due to the following two reasons. On the one hand, the electrostatic interaction and π-π interaction between MB and MWCNT endowed the MWCNT-MB composite with good stability. On the other hand, the coated PDA layer can further stabilize the components in the inner layer.

**FIGURE 3:**
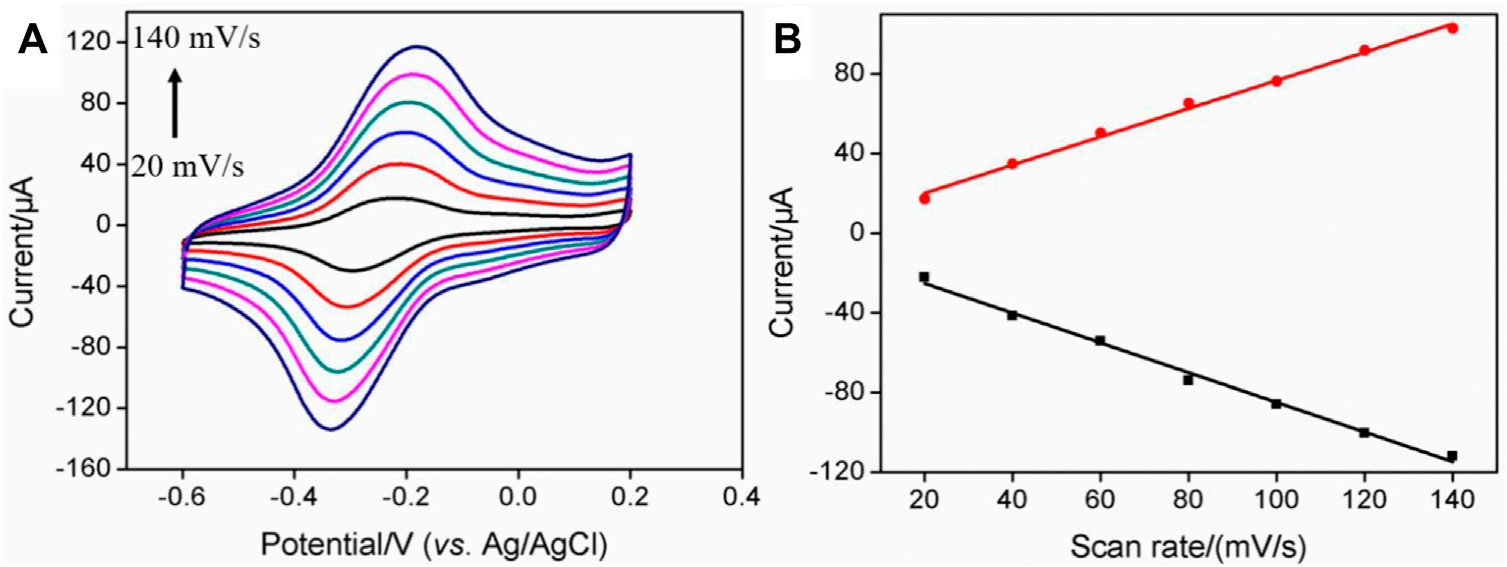
**(A)** CV curves of PDA/MWCNT-MB/GCE at different scan rate. The electrolyte solution is 0.1 M PBS (pH = 7.4). **(B)** The linear regression curve between the oxidation peak current or reduction peak current and scan rate.

The electrochemical behaviors of MB on the electrode surface were further investigated at different scan rates. As shown in Figure 3A, the redox peak currents of MB increased with the increase in scan rate. Linear correlations between the anodic peak current (*I*_pa_) or cathodic peak current (*I*_pc_) and scan rate (*v*) were revealed (Figure 3B, *I*_pa_ = 0.709 *v* + 5.86, R^2^ = 0.995, *I*_pc_ = -0.747 *v*-10.3, R^2^ = 0.993), demonstrating a characteristic surface-controlled electrochemical process of the immobilized probe.

**FIGURE 4:**
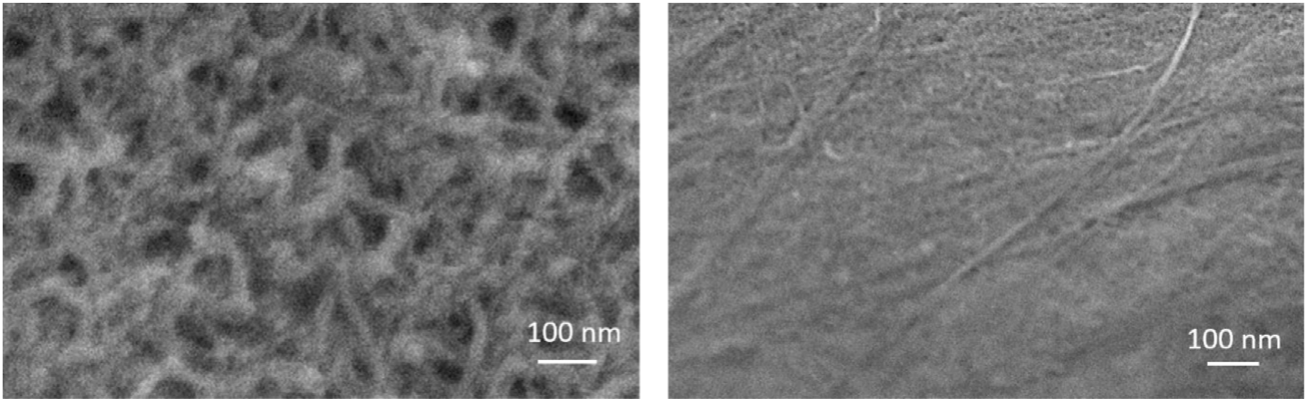
SEM images of MWCNT-MB **(left)** and PDA/MWCNT **(right)** modified glassy carbon sheet.

The morphology of MWCNT-MB/GCE and PDA/MWCNT-MB/GCE were investigated using a scanning electron microscopy (SEM). The left image in Figure 4 displayed the rough surface of MWCNT-MB/GCE with uniform distribution of MWCNT. The characteristics signals including C and O elements mainly form MWCNT, and N and S elements from MB in SEM-energy dispersive spectroscopy (SEM-EDS) also prove the successful modification of electrodes with MWCNT-MB nanocomposites. After the coating of PDA, the rough and porous network of carbon nanotubes was not obvious, indicating the effective coverage of the PDA layer (the right image in Figure 4).

### Optimized conditions for the fabrication of immunosensor

**FIGURE 5:**
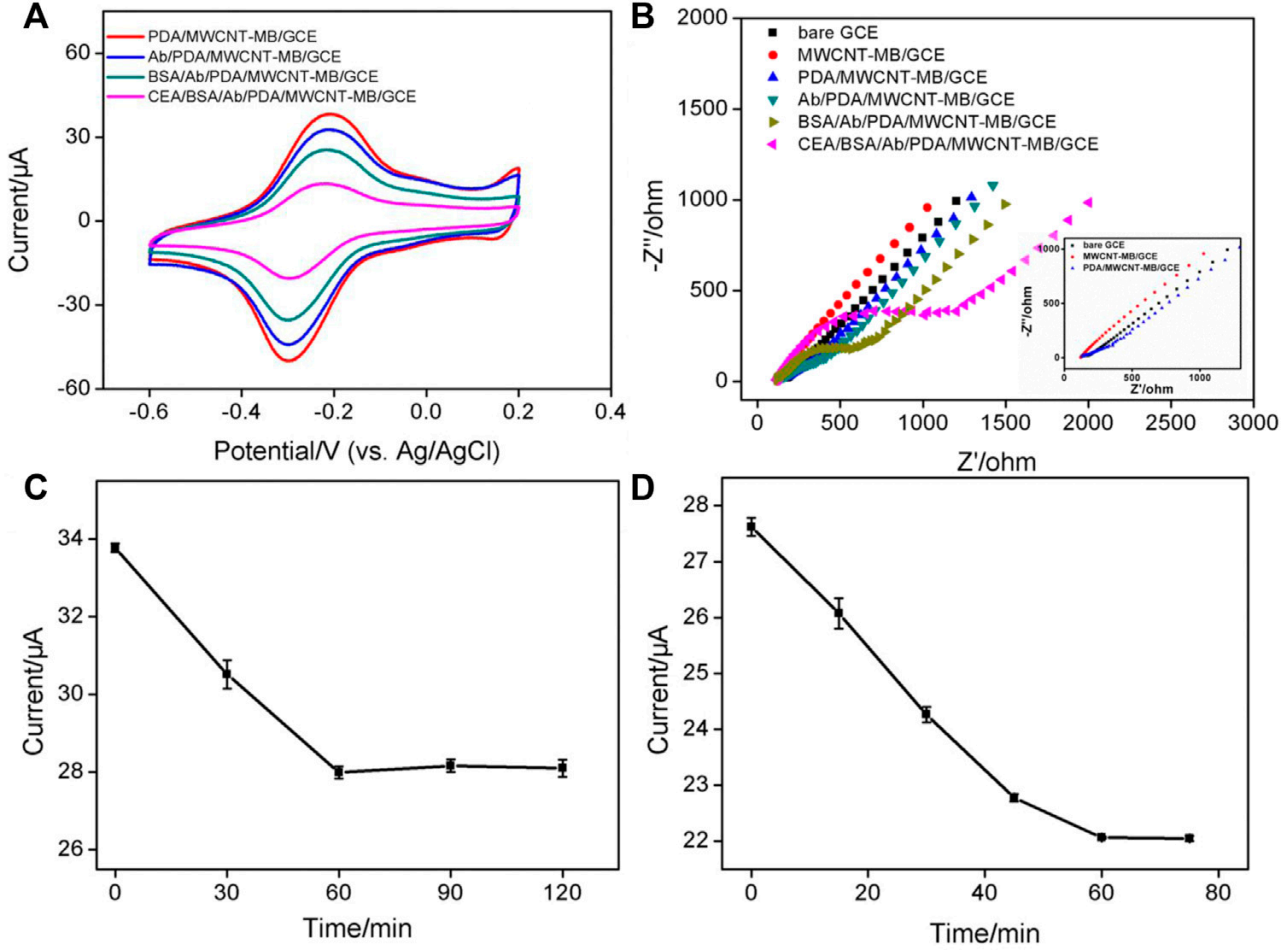
**(A)** CV obtained on different electrodes. **(B)** Nyquist plots obtained in KCl (0.1 M) containing Fe(CN)_6_^3/4−^ (2.5 mM). Inset is the magnified Nyquist plots, respectively. **(C)** The peak current obtained after CEA binding on different immunosensors fabricated using different reaction time between PDA and Ab. **(D)** The peak current obtained after CEA binding using different incubation time between CEA and Ab. The concentration of Ab for the fabrication of the immunosensor is 100 μg/ml. The used concentration of CEA is 1 ng/ml.

Figure 5 presents the CV curves obtained on different electrodes during antibody binding. The dopaquinone structure in PDA can react with −NH_2_ or −SH groups of proteins to achieve covalent immobilization of antibodies. As seen, the electrochemical signal of MB on Ab/MWCNT-MB/GCE significantly reduced after Ab binding (Figure 5A). This was due to the insulating properties of the protein, which hindered the interfacial electron transfer. After subsequent blocking of non-specific sites with BSA, the immunosensor BSA/Ab/MWCNT-MB/GCE is finally obtained. This blocking process further leads to a decrease in the peak current of immobilized MB. When the constructed immunosensor is further incubated with CEA, the electrochemical signal of MB is remarkably reduced, suggesting the specific binding of antigen to antibody. The changes in the electrode surface during the construction of the immunosensor were also characterized by electrochemical impedance spectroscopy (EIS, Figure 5B). It is well known that the charge transfer resistance (*R*_ct_) of an electrode is related to the diameter of the semicircle of the Nyquist plots in the high-frequency region. (Ashraf et al., 2020; Iftikhar et al., 2021). In comparison with that of bare GCE (*R*_ct_ of 128 Ω), the *R*_ct_ of MWCNT-MB/GCE decreased (42 Ω) resulting from the good electronic conductivity of MWCNT. The *in-situ* formation of the PDA layer increased the *R*_ct_ of the PDA/MWCNT/GCE electrode (144 Ω) because of the non-conductive property of PDA. During the subsequent covalent immobilization of Ab followed by BSA blocking, the *R*_ct_ of immunosensor (BSA/Ab/PDA/MWCNT/GCE) became larger (410 Ω) because the protein layer acted as an inert layer and hindered electron transfer. When the fabricated immunosensor was incubated with CEA, a significant increase in impedance values (634 Ω) was observed, indicating successful capture of antigen at the immuno-recognitive interface. Randles-Sevcik equation is used to calculate the electrochemically active surface area (EASA) of different electrodes as follows:

Ip=(2.69×105)n32ACD12v12, (1)

where n, A, C, D, and *v* are the number of electron transfer, electrode area, the molecular concentration of redox solution, diffusion coefficient, and scan rate respectively (He et al., 2015; Asif et al., 2019; Alam and Deen, 2020; Ashraf et al., 2022b) The calculated EASA of MWCNT-MB/GCE (0.346 cm^2^) notably increased in comparison with that of bare GCE (0.0584 cm^2^), owing to the generation of conductive MWCNT network. On the other hand, the EASA of electrodes obtained after the growth of PDA (0.307 cm^2^ for PDA/MWCNT/GCE) or the fabrication of immuno-recognitive interface (0.202 cm^2^ for BSA/Ab/PDA/MWCNT/GCE) gradually decreased because of the coverage of non-conductive substances.

To achieve the best detection sensitivity, the parameters for the construction of the immunosensor include the applied concentrations of MWCNT and dopamine, the reaction time between Ab and PDA, and the incubation time for the binding of antigen, and antibody, and the used concentration of Ab are optimized. When the concentration of MB was fixed, too low a concentration of MWCNT resulted in a low peak current of MB on the electrode due to less amount of bound MB (Supplementary Figure S3 in SI). The highest peak current was obtained when the concentration of MWCNT was 0.5 mg/ml. Then, even though the concentration of MWCNT continued to increase, the current value of MB decreased resulting in the decreased water dispersibility of MWCNT. Thus, the concentration of MWCNT was set as 0.5 mg/mL. As revealed in Supplementary Figure S4 (SI), the used concentration of dopamine had little effect on the electrochemical response of the electrode. High concentrations of dopamine only resulted in a weak decrease of the current signal, which was attributed to the larger deposition amount of non-conductive PDA. The chosen dopamine concentration was 0.38 mg/ml. Figure 5C shows the peak currents of MB obtained on immunosensor that are fabricated using different reaction time between Ab and PDA. As revealed, the current of MB decreased with the increase of reaction time and then reached a stable plateau when the reaction time was 60 min. This indicated that the immobilization of the antibody was saturated at 60 min. Therefore, the reaction time between Ab and PDA layer was chosen to be 60 min for further investigation. Figure 5D presents the electrochemical signals of MB obtained under different incubation times of antigen and antibody. When the incubation time was too short, the reaction equilibrium of antigen and antibody can not be reached, leading to insufficient reduction of MB signal. When the incubation time reached 1 h, further increasing the incubation time did not cause changes in the current signal of MB, suggesting a reaction balance between antigen and antibody. Thus, the incubation time between antigen and antibody was set as 1 h for the subsequent experiments. The binding amount of the Ab on the electrode surface reached saturation when the used concentration of Ab was 100 μg/ml (Supplementary Figure S5 in SI). In addition, the effect of pH of the electrochemical support solution was also studied. As shown in Supplementary Figure S6 (in SI), the current signal of MB after CEA binding decreased with increasing pH. This was due to the participation of protons in the electrochemical redox process of MB. As the pH value increased, the H^+^ concentration in the solution decreased, which was not beneficial for the electrochemical oxidation of MB, resulting in a decreased peak current (He et al., 2015; Yang et al., 2018).

### Reagentless electrochemical determination of carcinoembryonic antigen

**FIGURE 6:**
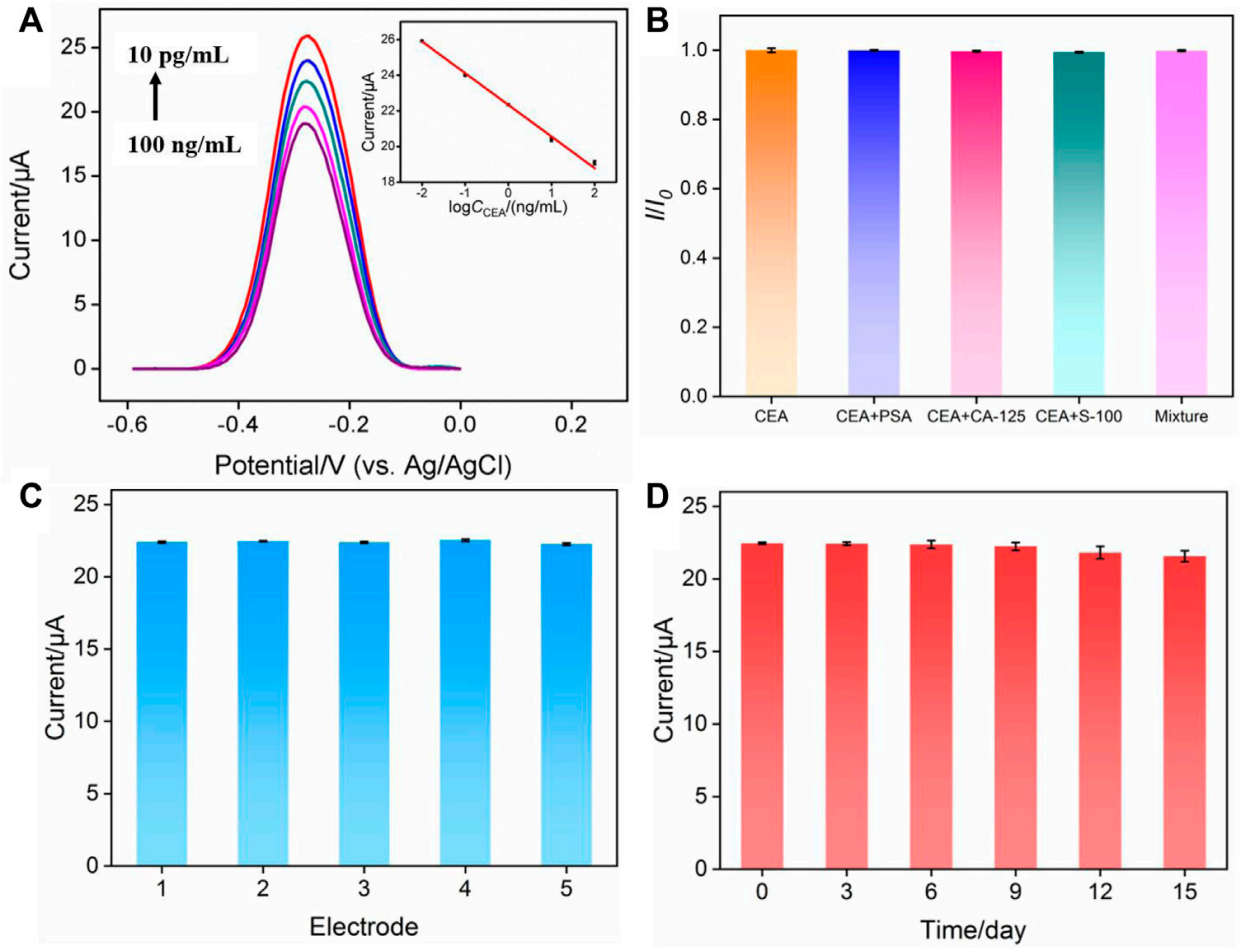
**(A)** Electrochemical response of the immunosensor in presence of different concentrations of CEA. Inset is the calibration curve for the determination of CEA. **(B)** Relative ratio of peak current before (*I*_0_) and after (*I*) incubation with CEA, CEA + PSA, CEA + CA125, CEA + S-100 or the mixture containing CEA, PSA, CA125, S-100. The concentration of CEA and other proteins are 0.1 ng/ml and 10 ng/ml, respectively. **(C)** The peak current obtained using five different electrodes. **(D)** The peak current obtained using electrodes stored for different time.

Based on the decrease of the electrochemical signal of MB after CEA binding with Ab, the fabricated immunosensor was applied for reagentless detection of CEA. After incubating the immunosensors with different concentrations of CEA, differential pulse voltammetry is used to detect the signal of MB on the electrode. The DPV curves are shown in Figure 6A. When the concentration of CEA ranged from 10 pg/ml to 100 ng/ml, as shown in the inset in Figure 6A, the peak current of MB (*I*) had a good linear relationship with the logarithm of CEA concentration (lg*C*_CEA_) (*I* = -1.77 lg*C*_CEA_ + 22.33, *R* ^2^ = 0.997). The limit of detection (LOD) based on three times signal-to-noise ratio (S/N = 3) was 0.6 pg/ml, where the signal was calculated as 3 times of noise and represented the decreased current resulting from the binding of CEA. The noise was the standard deviation (SD) of the blank current of the immunosensor before incubation of CEA. Supplementary Table S1 (SI) shows the comparison of the detection of CEA using different methods (Li et al., 2017; Barman et al., 2018; Han et al., 2018; Ma et al., 2019; Li et al., 2020; Tang et al., 2020; Wu et al., 2021). The LOD is lower than that obtained using nanopipette analysis based on CEA aptamer modified magnetic Fe_3_O_4_-Au nanoparticles (Apt-MNPs), (Tang et al., 2020), or fluorescence determination using CEA aptamer modified Cu^2+^-loaded UiO-66 metal-organic framework (Cu-UiO-66/CEA-Apt) (Wu et al., 2021), or photoelectrochemical determination based on WO_3_@BiOI modified indium-tin oxide electrode followed with loading CdS NWs (CdS@BiOI@WO_3_/ITO) (Han et al., 2018), or electrochemical determination based on immobilization of Ab on poly(ethyleneglycol)-NH_2_ connected pyrenebutyric acid functionalized graphene/gold nanoparticles (BSA/Ab/AuNPs/PPYGR/GCE) (Li et al., 2017), and Pd@Au@Pt nanocomposite/-COOH terminated reduced graphene oxide (Barman et al., 2018). The LOD is higher than the that obtained using sandwich-type electrochemical determination based on trimetallic yolk-shell Au@AgPt nanocubes loaded on amino-functionalized MoS_2_ nanoflowers (MoS_2_ NFs/Au@AgPt YNCs) and Au triangular nanoprisms (Au TNPs) (Ma et al., 2019), and immunointerface using NH_2_-functionalized CEA aptamer on Fe-MOF modified with self-polymerized dopamine-decorated Au (NH_2_-aptamer/Au@PDA@Fe-MOF/GCE) (Li et al., 2020).

### Selectivity, reproducibility, and stability of the fabricated immunosensor

Selectivity is important to evaluate the performance of immunosensors. The selectivity of the fabricated immunosensor was examined by performing detection of CEA in the absence or presence of one or the mixture of other tumor markers including prostate-specific antigen (PSA), and carcinoma antigen 125 (CA125), and calcium-binding protein β (S-100). As shown in Figure 6B, both one or several other tumor markers almost had no effect on the detection of CEA, proving the specificity of the constructed immuno-recognitive interface and the selectivity of the immunosensor. To evaluate the reproducibility of the fabricated immunosensor, five electrodes were prepared in parallel. The relative standard deviation (RSD) for the detection of CEA was 0.5%, indicating high reproducibility (Figure 6C). When electrodes were stored at 4°C for 15 days, the response of the immunosensor towards CEA remained at 98.3% of the initial signal, demonstrating the high stability of the sensor (Figure 6C).

### Real sample analysis

The feasibility of immunosensors in the analysis of the real sample is the prerequisite for their clinical application. To investigate the detection reliability of the sensor, determination of CEA in human serum was performed. The CEA concentration values determined in a human serum sample (a healthy woman) by the proposed immunosensor and ROCHE ELISA electrochemiluminescence analyzer were 2.10 ± 0.08 ng/ml (mean ± SD, *n* = 3) and 2.18 ± 0.05 ng/ml (mean ± SD, *n* = 3), respectively. F-test and t-test were performed to assess whether there was statistical significance between the two means. The obtained F-value (2.56) was lower than the critical F-value (19.00), suggesting that the precision of the two data was not significantly different from each other. The obtained t-value (1.47) was lower than the t-critical two-tail value (2.13), indicating that there was no statistical significance between the two methods.

## Conclusion

In summary, a simple and convenient electrochemical immunosensor was constructed to realize rapid, highly sensitive, and reagentless detection of the tumor marker, carcinoembryonic antigen. To realize direct detection of CEA without adding additional solution-based electrochemical probes, the redox probe methylene blue (MB), which had distinguished potential with the common electroactive interferences in biological samples, was immobilized on the surface of the electrode. To increase the active area and conductivity of the electrode, a multi-walled carbon nanotube composite was employed as an electronic wire to improve the performance of the development. Efficient immobilization of the antibody was achieved by an *in-situ* coating of polydopamine layer, which acts as the linkage to covalently immobilize the antibody. Based on the decrease of the electrochemical signal of confined MB after the binding of antigen, the fabricated immunosensor can achieve reagentless and sensitive detection of CEA. As the electrode interface simultaneously contains electron transfer wires, confined redox probes, and effectively immobilized recognition antibodies, the fabricated immunosensor exhibits simple preparation, sensitive detection, and good selectivity, demonstrating great potential in the sensitive detection of tumor markers.

## Data Availability

The original contributions presented in the study are included in the article/Supplementary Material; further inquiries can be directed to the corresponding author.
