# Supplementary material for: A reagentless electrochemical immunosensor for sensitive detection of carcinoembryonic antigen based on the interface with redox probe-modified electron transfer wires and effectively immobilized antibody
Source: Front Chem. 2022 Aug 8;10:939736. doi: 10.3389/fchem.2022.939736 (PMC9393226; doi:10.3389/fchem.2022.939736)
Supplement: Supplementary file 1 [file Table1.DOC]

TABLES

Table S1. Comparison between detection of CEA using different method.

| Materials | Method | Liner range ng/mL | LOD  pg/mL | Ref. |
| --- | --- | --- | --- | --- |
| CEA-Apt/MNPs | Nanopipette analysis | 2-200 | 600 | 56 |
| Cu-UiO-66 MOF/CEA-Apt | Fluoresence | 0.01-0.3 | 10 | 57 |
| Ab/CdS@BiOI  @WO_3_/ITO | Photoelectrochemical | 0.01-50 | 3.2 | 58 |
| MoS_2_NFs/Au@AgPtYNCs-Ab_2_/CEA/BSA/Ab_1_/  AuTNPs/GCE | Amperometric | 10^-6^-100 | 3.09×10^-3^ | 59 |
| BSA/NH_2_-aptamer/Au@PDA@Fe-MOF/GCE | DPV | 10^-6^-10^3^ | 3.3×10^-4^ | 60 |
| BSA/Ab/AuNPs/PPYGR/GCE | EIS | 0.1-10^3^ | 60 | 61 |
| Ab/PdAuPt/  COOH-rGO/Au | DPV | 0.005-50 | 1 | 62 |
| BSA/Ab/PDA/MWCNT-MB/GCE | DPV | 0.01-100 | 0.55 | Our work |

Apt, Aptamer; MNPs, magnetic Fe_3_O_4_-Au nanoparticles; Cu-UiO-66 MOF, UiO-66 metal-organic framework loaded with Cu^2+^; CdS, cadmium sulfide; BiOI, bismuth oxyiodide; WO_3_, tungstic anhydride; ITO, indium-tin oxide; MoS_2_NFs, MoS_2_ nanoflowers; Au@AgPt YNCs, trimetallic yolk-shell Au@AgPt nanocubes; Ab_2_, secondary antibody; Au TNPs, Au triangular nanoprisms; MOF, metal-organic frameworks; AuNPs, gold nanoparticles; PPYGR, poly(ethyleneglycol)-NH_2_/pyrenebutyric acid functionalized graphene; EIS, Electrochemical impedance spectroscopy; PdAuPt, Pd@Au@Pt nanocomposites; COOH-rGO, -COOH terminated reduced graphene oxide.

FIGURES


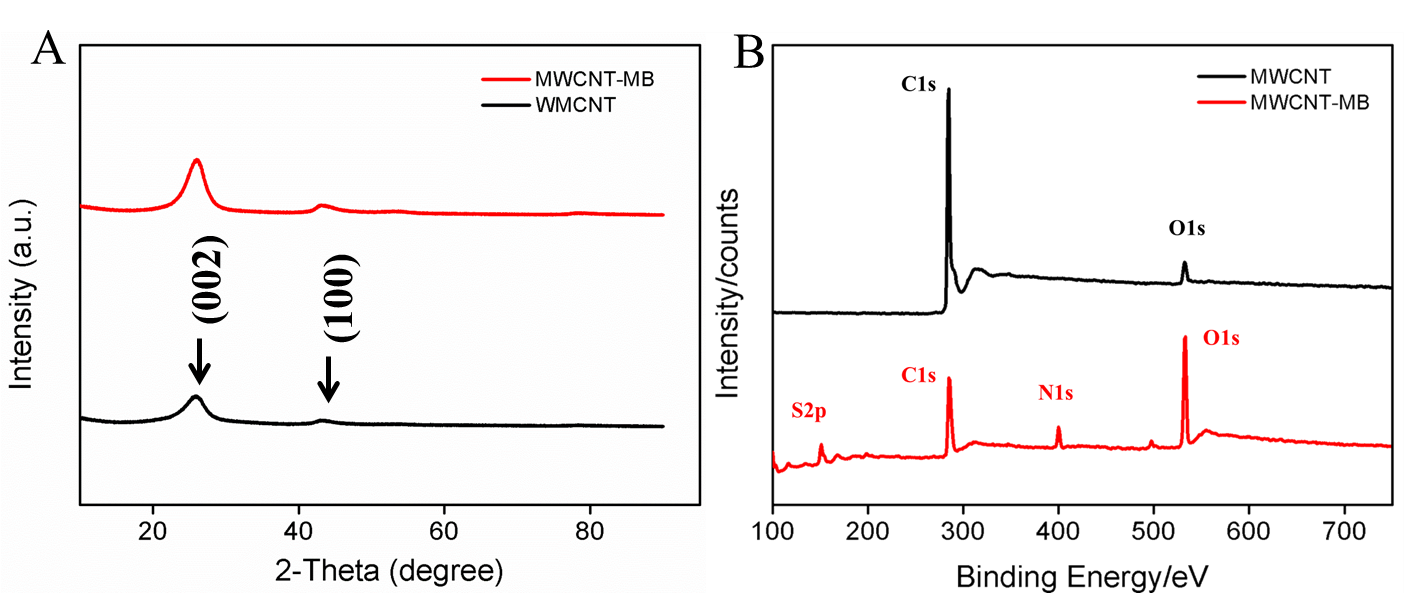


**Figure S1** XRD patterns (A) and XPS survey spectrum (B) of MWCNT and MWCNT-MB.


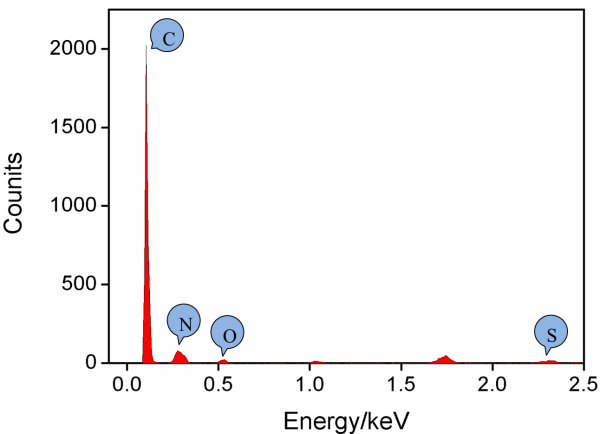


**Figure S2** SEM-EDS of MWCNT-MB/GCE electrode.


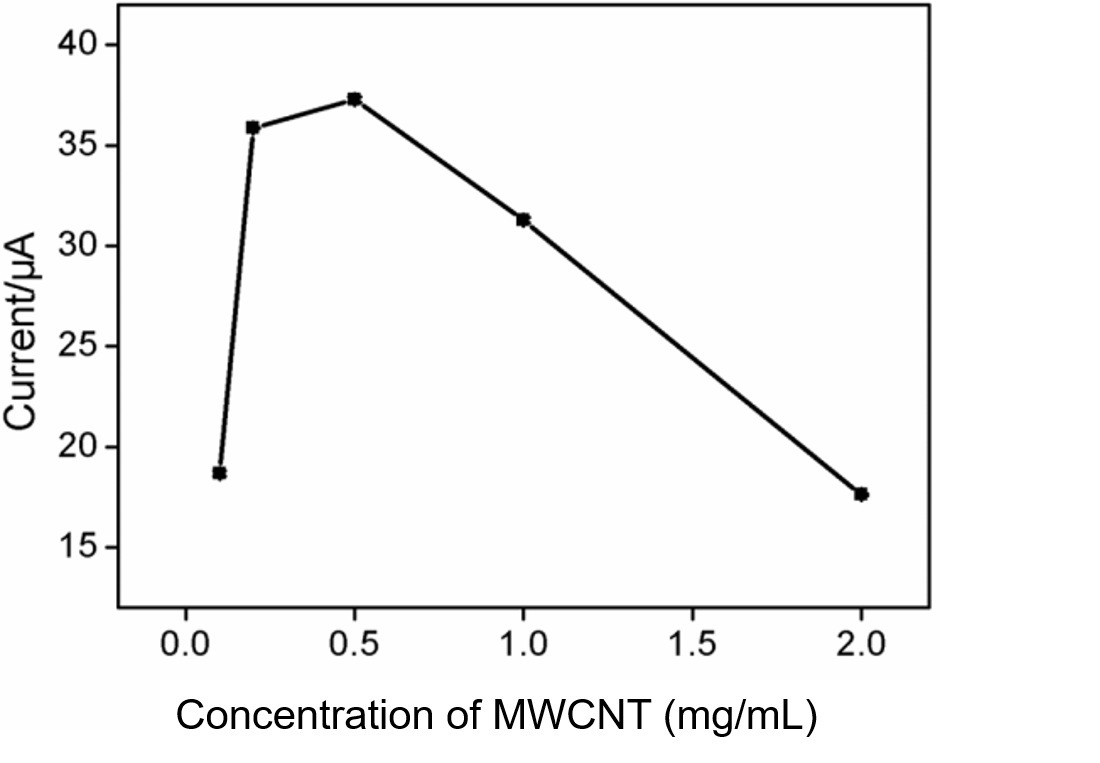


**Figure S3** The peak current obtained after CEA binding on different immunosensors fabricated using different concentration of MWCNT.


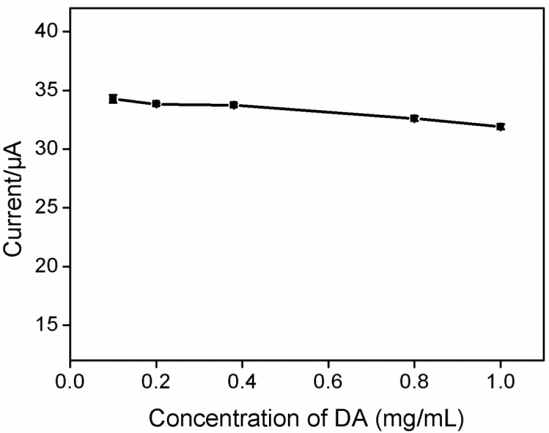


**Figure S4** The peak current obtained after CEA binding on different immunosensors fabricated using different concentration of dopamine.


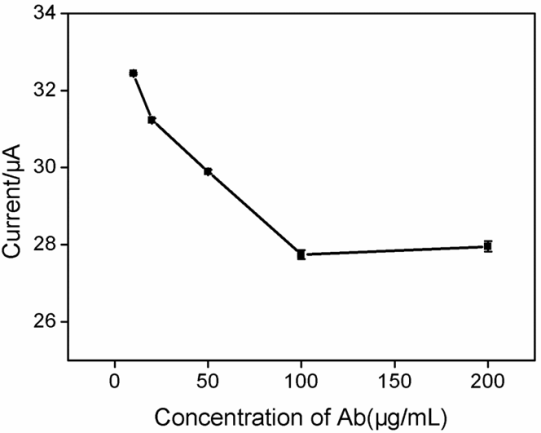


**Figure S5** The peak current obtained after CEA binding on different immunosensors fabricated using different concentration of Ab.


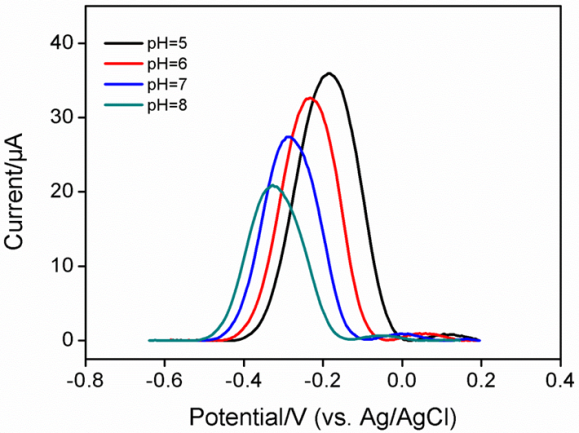


**Figure S6** The peak current obtained after CEA binding in PBS with different pH.
