# Supplementary material for: A reagentless electrochemical immunosensor for sensitive detection of carcinoembryonic antigen based on the interface with redox probe-modified electron transfer wires and effectively immobilized antibody
Source: Front Chem. 2022 Aug 8;10:939736. doi: 10.3389/fchem.2022.939736 (PMC9393226; doi:10.3389/fchem.2022.939736)
Supplement: Supplementary file 2 [file DataSheet1.PDF]

TABLES

Table S1. Comparison between detection of CEA using different method.

Materials	Method	Liner range ng/mL	LOD pg/mL	Ref.
CEA-Apt/MNPs	Nanopipette analysis	2-200	600	56
Cu-UiO-66 MOF/CEA-Apt	Fluoresence	0.01-0.3	10	57
Ab/CdS@BiOI @WO_3/ITO	Photoelectrochemical	0.01-50	3.2	58
MoS_2NFs/Au@AgPtYNCs-Ab_2/CEA/BSA/Ab_1/ AuTNPs/GCE	Amperometric	10^{-6}-100	3.09×10^{-3}	59
BSA/NH_2-aptamer/Au@PDA@Fe-MOF/GCE	DPV	10^{-6}-10^{3}	3.3×10^{-4}	60
BSA/Ab/AuNPs/PPYGR/GCE	EIS	0.1-10^{3}	60	61
Ab/PdAuPt/ COOH-rGO/Au	DPV	0.005-50	1	62
BSA/Ab/PDA/MWCNT-MB/GCE	DPV	0.01-100	0.55	Our work

Apt, Aptamer; MNPs, magnetic Fe_3O_4-Au nanoparticles; Cu-UiO-66 MOF, UiO-66 metal-organic framework loaded with Cu^{2+}; CdS, cadmium sulfide; BiOI, bismuth oxyiodide; WO_3, tungstic anhydride; ITO, indium-tin oxide; MoS_2NFs, MoS_2 nanoflowers; Au@AgPt YNCs, trimetallic yolk-shell Au@AgPt nanocubes; Ab_2, secondary antibody; Au TNPs, Au triangular nanoprisms; MOF, metal-organic frameworks; AuNPs, gold nanoparticles; PPYGR, poly(ethyleneglycol)-NH_2/pyrenebutyric acid functionalized graphene; EIS, Electrochemical impedance spectroscopy; PdAuPt, Pd@Au@Pt nanocomposites; COOH-rGO, -COOH terminated reduced graphene oxide.

FIGURES

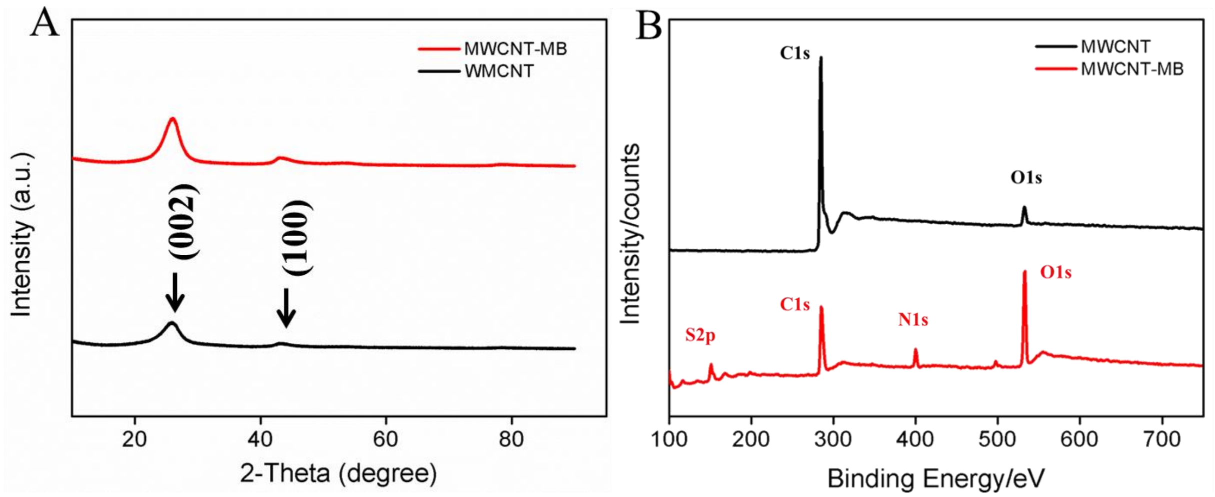

Figure S1 XRD patterns (A) and XPS survey spectrum (B) of MWCNT and MWCNT-MB.

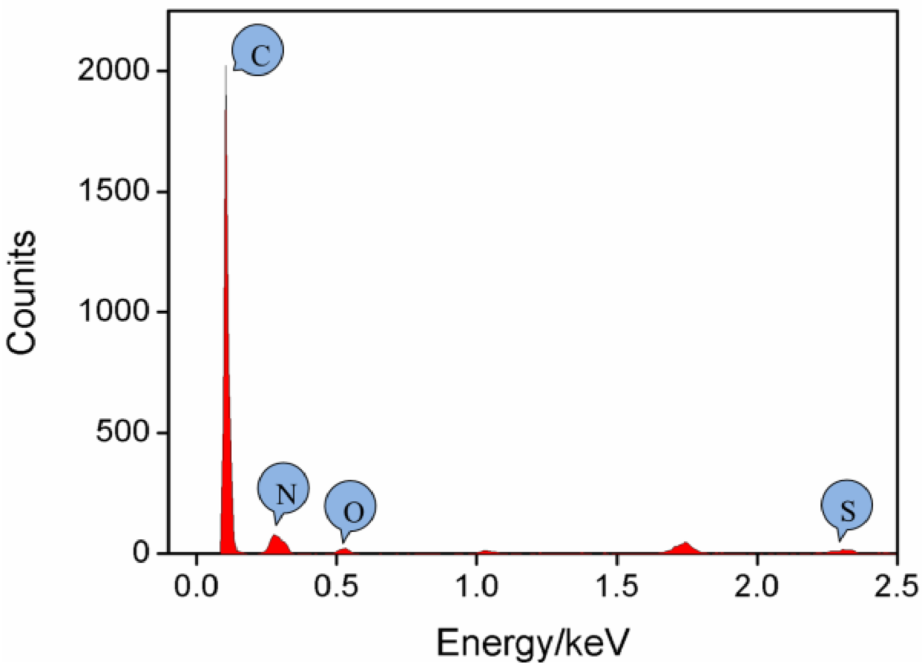

Figure S2 SEM-EDS of MWCNT-MB/GCE electrode.

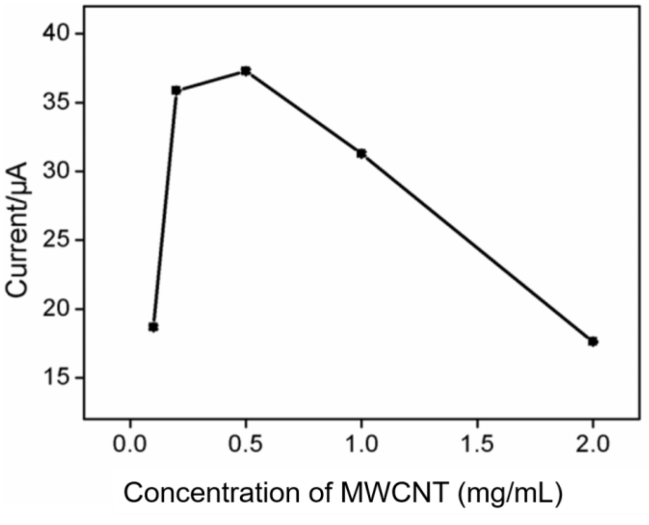

Figure S3 The peak current obtained after CEA binding on different immunosensors fabricated using different concentration of MWCNT.

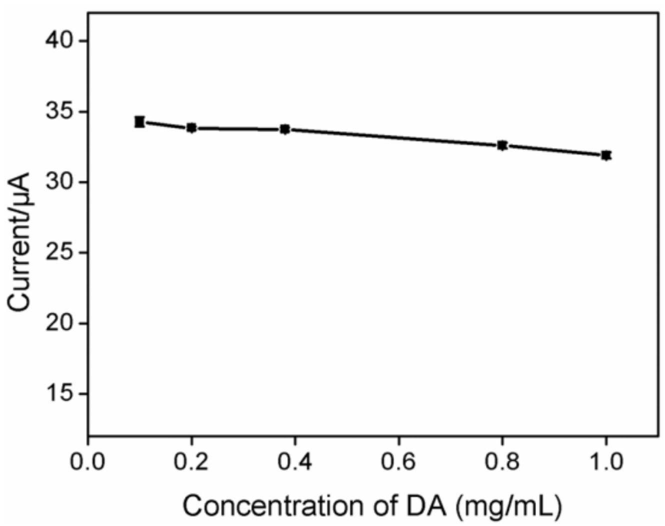

Figure S4 The peak current obtained after CEA binding on different immunosensors fabricated using different concentration of dopamine.

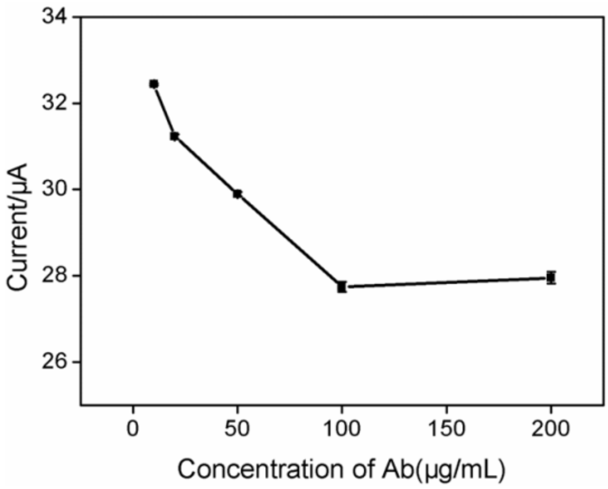

Figure S5 The peak current obtained after CEA binding on different immunosensors fabricated using different concentration of Ab.

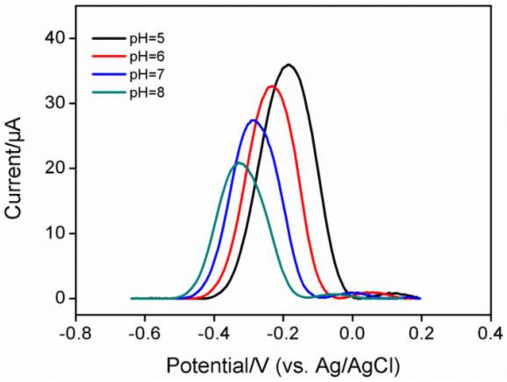

Figure S6 The peak current obtained after CEA binding in PBS with different pH.
